# Identification of candidate structured RNAs in the marine organism '*Candidatus *Pelagibacter ubique'

**DOI:** 10.1186/1471-2164-10-268

**Published:** 2009-06-16

**Authors:** Michelle M Meyer, Tyler D Ames, Daniel P Smith, Zasha Weinberg, Michael S Schwalbach, Stephen J Giovannoni, Ronald R Breaker

**Affiliations:** 1 Department of Molecular Cellular and Developmental Biology, Yale University, Box 208103, New Haven, CT 06520, USA; 2 Department of Molecular Biophysics and Biochemistry, Yale University, Box 208103, New Haven, CT 06520, USA; 3 Howard Hughes Medical Institute, Yale University, Box 208103, New Haven, CT 06520, USA; 4 Department of Microbiology, Oregon State University, Corvallis, OR 97333, USA

## Abstract

**Background:**

Metagenomic sequence data are proving to be a vast resource for the discovery of biological components. Yet analysis of this data to identify functional RNAs lags behind efforts to characterize protein diversity. The genome of '*Candidatus *Pelagibacter ubique' HTCC 1062 is the closest match for approximately 20% of marine metagenomic sequence reads. It is also small, contains little non-coding DNA, and has strikingly low GC content.

**Results:**

To aid the discovery of RNA motifs within the marine metagenome we exploited the genomic properties of '*Cand*. P. ubique' by targeting our search to long intergenic regions (IGRs) with relatively high GC content. Analysis of known RNAs (rRNA, tRNA, riboswitches etc.) shows that structured RNAs are significantly enriched in such IGRs. To identify additional candidate structured RNAs, we examined other IGRs with similar characteristics from '*Cand*. P. ubique' using comparative genomics approaches in conjunction with marine metagenomic data. Employing this strategy, we discovered four candidate structured RNAs including a new riboswitch class as well as three additional likely *cis*-regulatory elements that precede genes encoding ribosomal proteins S2 and S12, and the cytoplasmic protein component of the signal recognition particle. We also describe four additional potential RNA motifs with few or no examples occurring outside the metagenomic data.

**Conclusion:**

This work begins the process of identifying functional RNA motifs present in the metagenomic data and illustrates how existing completed genomes may be used to aid in this task.

## Background

The discovery of many RNA sequences that do not encode proteins (non-coding RNAs or ncRNA) and have biological functions beyond those of tRNA and rRNA, has significantly expanded the known role of RNA in diverse cellular processes. Consequently, there is a growing effort to systematically identify ncRNAs utilizing both experimental and computational techniques. Experimental approaches are typically used to identify non-coding portions of an organism's genome that are actively being transcribed. These approaches are not dependent on the identification of conserved RNA sequences or secondary structures, and therefore are well-suited for the discovery of unstructured or poorly-conserved ncRNAs. However, experimental limitations can cause some RNAs to be missed, and the false-positive rate may be high due to "transcriptional noise" [1,2]. Alternatively, computational methods seek to identify evidence of conserved RNA sequences and secondary structures through comparative genomics [3,4]. However, such methods usually cannot be used to identify RNA motifs that may not have conserved secondary structure, are small with few base-pairing elements, or are not well-represented in genomic sequence databases.

Marine metagenomic sequence data are a proven resource for the discovery of novel protein diversity and have provided additional examples for thousands of previously identified open reading frames (ORFs) with no known homologs [5]. While there have been surveys conducted with the marine metagenome to discover additional examples of known ncRNAs [6,7], there have been no studies explicitly examining these data for novel RNA motifs, in part due to unique computational challenges inherent to metagenomic datasets. Specifically, the exceedingly large amount of sequence data available (~7 billion base pairs), relatively poor annotation of protein coding regions due to a high frequency of fragmentary genes that result from short sequence reads, and comparatively high sequencing error rates make metagenomic data analysis difficult [8-10].

To circumvent many of the challenges associated with analyzing metagenomic sequence data, we have used the genome of '*Cand*. P. ubique' HTCC 1062 as a starting point to discover new RNA motifs within the marine metagenome. Bacteria of the SAR11 clade, of which '*Cand*. P. ubique' is a representative, are found throughout the world's oceans and are the dominant aerobic heterotrophs in marine surface waters [11]. Given its numeric advantage, genes from members of the SAR11 clade are well-represented in marine metagenomic libraries with nearly 20% of sequence reads from the Global Oceanographic Survey (GOS) matching most closely to genes present in the '*Cand*. P. ubique' genome [12,13]. Only ~30% of the GOS reads could be aligned well to the 584 available reference genomes. The other predominant genera represented in the GOS data are *Prochlorococcus, Synechococcus, Burkholderia*, and *Shewanella*, none of which are closely related to '*Cand*. P. ubique'. While, alignments to every reference genome were identified, typically they showed identity to regions corresponding to large, highly conserved genes [13].

At 1.3 million base pairs, the genome of '*Cand*. P. ubique' is the smallest known for a free-living organism, but it appears to encode for nearly all the basic functions of Alphaproteobacteria cells [14]. The genome contains very little non-coding DNA, with a median intergenic region (IGR) length of 3 nucleotides. In addition, the organism has remarkably low GC content (29%). While evaluating nucleotide composition is usually not a viable method for identifying ncRNAs [15], in genomes with a strong AT bias or hyperthermophilic environment, the higher GC content necessary to maintain a stable RNA structure may be used to identify candidate ncRNAs [16-19]. '*Cand*. P. ubique' offers an ideal opportunity to utilize nucleotide composition as its genome has very few long IGRs, which are generally low GC (23% on average).

In the current study we combine nucleotide composition with comparative genomics approaches to identify novel structured RNA motifs in '*Cand*. P. ubique' and the marine metagenomic data. First, we demonstrate that longer, higher GC '*Cand*. P. ubique' IGRs are much more likely to contain structured RNAs (rRNAs, tRNAs, etc.). Subsequently, we utilized the IGRs in '*Cand*. P. ubique' with similar properties that lack assigned ncRNAs as the starting point for a comparative sequence analysis strategy that takes advantage of marine metagenomic sequences. We discovered four likely structured ncRNAs including a new riboswitch class, and three other candidate *cis*-regulatory motifs. In addition we describe several other conserved IGRs that encode potential structured RNA elements.

## Results

### Analysis strategy

**Figure 1:**
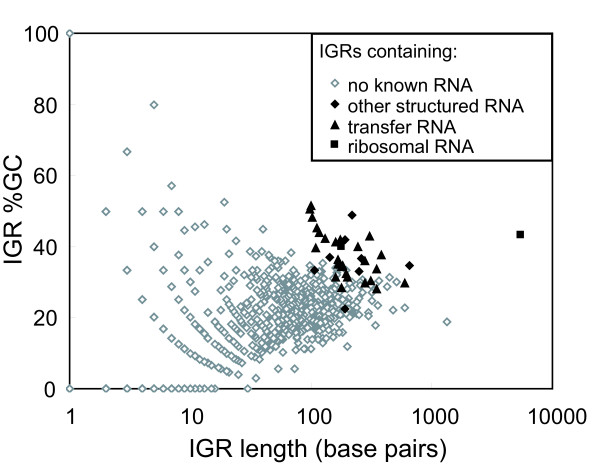
**Percent GC-content versus length of intergenic regions (IGRs) in '*Cand*. P. ubique'**. Transfer and ribosomal RNAs are as annotated by Rfam [24] and RefSeq (RefSeq accession NC_007205.1). Other structured RNAs include known riboswitches, 4.5S RNA (SRP RNA), RNase P RNA and tmRNA.

To identify potential ncRNAs in the genome of '*Cand*. P. ubique', all IGRs were extracted from the '*Cand*. P. ubique' genome and ranked by GC content. When '*Cand*. P. ubique' IGRs are plotted by their length and percent GC, those containing annotated RNAs (rRNAs, tRNAs, riboswitches, etc.) cluster toward the top right of the graph (Figure 1). This finding indicates that the vast majority of GC-enriched IGRs longer than 100 bp carry annotated ncRNAs (Additional file 1).

To identify additional structured RNAs that may not be annotated, we performed BLAST searches of the remaining IGRs against the Community Cyberinfrastructure for Advanced Marine Microbial Ecology Research and Analysis (CAMERA) database [20]. Table 1 lists GC enriched '*Cand*. P. ubique' IGRs longer than 100 bp and the number of BLAST hits identified with an E-value less than 10^-5 ^as a measure of conservation. The average number of blast hits for IGRs containing tRNAs is 2158, with a standard deviation of 1282. However, the average number of blast hits for the '*Cand*. P. ubique' IGRs containing SAM-II riboswitches, which are significantly smaller than a tRNA and most commonly present in Alpha-, Beta- and Gammaproteobacteria, is approximately 500. Based on this analysis and the need for a relatively large number of BLAST hits for subsequent comparative sequence analysis algorithms, IGRs with greater than 200 BLAST hits were further screened for unannotated ncRNAs and misannotated protein coding sequence. This screening process revealed several misannotated protein coding sequences in addition to several known structured RNAs not previously annotated (Additional file 2 – Table 1). The RNA motifs identified are typically very highly ranked on our list, and include tmRNA, the RNA component of the signal recognition particle (SRP), the RNase P RNA (class A), and a number of riboswitches (Table 1).

**Table 1:** '*Cand*. P. ubique' IGRs longer than 100 bp ranked by GC content. IGRs containing tRNA and rRNA removed

Coordinates	Length	%GC	BLAST Hits	RNA (strand)	Locus Tag	Flanking Gene Name (Strand)	Locus Tag	Flanking Gene Name (strand)
10302–10518	217	48.85	1761	tmRNA (+)	SAR11_0010	*thyX *(-)	SAR11_0011	COG4696 (-)
649763–649953	191	41.88	1990	glycine riboswitch (+)	SAR11_0664	membrane prot.(+)	SAR11_0666	gcvT (+)
493521–493664	144	36.81	888	4.5 S RNA (SRP RNA) (+)	SAR11_506	*pheA *(+)	SAR11_0507	*dnaX *(+)
1127293–1127553	261	36.78	127	SAM-II/SAM-V riboswitch (-)	SAR11_1129	*bhmt *(-)	SAR11_1730	hyp. protein (-)
564786–564910	125	35.2	611	*pntA *element (+)	SAR11_0573	*rpmJ *(+)	SAR11_0574	*pntA *(+)
38796–39447	652	34.51	2475	RNase P RNA (-)	SAR11_0033	*mraZ *(-)	SAR11_0034	*ybjR *(-)
260190–260348	159	33.96	1615	*ffh *motif (-)	SAR11_2356	*ffh *(-)	SAR11_0257	*dapF *(+)
626974–627168	195	33.33	1168		SAR11_0641	*recA *(+)	SAR11_0642	protease (-)
786467–786574	108	33.33	927	TPP riboswitch (+)	SAR11_0810	hyp. protein (+)	SAR11_0811	transporter (+)
585015–585135	121	33.06	41		SAR11_0599	COG1729 (+)	SAR11_0600	*mesj *(+)
498458–498706	249	32.93	2398	glycine riboswitch (-)	SAR11_0510	*glcB *(-)	SAR11_0511	*accA *(+)
622388–622552	165	32.73	1301	SAR11_0636 element (+)	SAR11_0635	hyp. protein (-)	SAR11_0636	hyp protein (+)
1142870–1143031	162	32.1	29		SAR11_1190	COG0659 (-)	SAR11_1191	HIT protein (-)
159067–159166	100	32	25		SAR11_0156	hyp. protein (-)	SAR11_0157	*ispA *(-)
1292813–1292925	113	31.86	57		SAR11_1357	*livF2 *(-)	SAR11_1358	*livG2 *(-)
1120412–1120856	445	31.46	66		SAR11_1164	lipoprotein (-)	SAR11_1165	exonuclease (+)
873155–873283	129	31.01	832	*rpsB *motif (+)	SAR11_0906	*dnaE *(+)	SAR11_0907	*rpsB *(+)
628285–628539	255	30.2	571		SAR11_0642	protease (-)	SAR11_0643	*alaS *(+)
1005679–1005890	212	30.19	483	SAM-V (+)	SAR11_1029	*rplM *(-)	SAR11_1030	*metY *(+)
361353–361571	219	30.14	76		SAR11_0369	*grpE *(-)	SAR11_0370	HAM1-like prot. (+)
1125490–1125606	117	29.91	11		SAR11_1171	*ordL *(-)	SAR11_1172	*osmC *(-)
1189853–1189956	104	29.81	25		SAR11_1248	hyp. protein (+)	SAR11_1249	hyp. protein (+)
676100–676308	208	28.7	193		SAR11_0691	hyp. protein (-)	SAR11_0692	*yajQ *(-)
1212757–1212865	109	29.36	22		SAR11_1279	membrane prot. (-)	SAR11_1280	hyp. protein (+)
732778–732938	161	29.19	446	SAM-V (-)	SAR11_0750	*mmuM *(-)	SAR11_0751	hyp. protein. (-)
57720–58035	316	29.11	25		SAR11_0046	autotransporter (-)	SAR11_0047	transcription regulator (+)
120095–120215	121	28.93	211	*bablM* element (+)	SAR11_0108	*rnhB *(+)	SAR11_0109	*babIM *(+)
762114–762332	219	28.31	55		SAR11_0784	hyp. protein (+)	SAR11_0785	hyp. protein (+)
834435–834636	202	28.22	42		SAR11_0864	hyp. protein (+)	SAR11_0865	transporter (+)
1164239–1164384	146	28.08	0		SAR11_1216	*ecpD *(+)	SAR11_1218	*sigB *(+)
52729–52884	157	28.02	22		SAR11_0042	autotransporter (-)	SAR11_0043	hyp. protein (-)
1297623–1297755	133	27.82	480	*rhtB *element (-)	SAR11_1362	*rhtB *(-)	SAR11_1363	hyp. protein (+)
675041–675166	126	27.78	205		SAR11_0690	hyp. protein (-)	SAR11_0691	hyp. protein (-)
762678–763012	335	27.76	76		SAR11_0785	hyp. protein (+)	SAR11_0786	*qacH *(-)
43688–43789	102	27.4	570		SAR11_0037	*rpoD *(-)	SAR11_0038	*dnaG *(-)
791867–792012	146	27.4	125		SAR11_0817	*hupA *(+)	SAR11_0818	*amtB (+)*
1132812–1132928	117	27.35	10		SAR11_1178	*pstC *(-)	SAR11_1179	*pstS *(-)
1123617–1123934	318	27.04	192		SAR11_1169	hyp. protein (-)	SAR11_1170	hyp. protein (-)
1181972–1182071	100	27	77		SAR11_1238	*sfuC *(-)	SAR11_1239	hyp. protein (-)
670506–670772	267	26.97	194		SAR11_0685	*moeA *(-)	SAR11_0686	hyp. protein (-)
1074189–1074359	171	26.9	650	*rpsL *motif (-)	SAR11_1121	*rpsL *(-)	SAR11_1122	*rpoC *(-)
164139–164261	123	26.82	90		SAR11_0160	COG0647G (-)	SAR11_0161	*groES (+)*
1245732–1245856	125	26.4	37		SAR11_1309	hyp. protein (+)	SAR11_1310	*amt *(+)

Identification of SRP RNA (4.5S RNA) [21] and RNase P RNA [22,23] was very straightforward. Both are completely contained within their respective IGRs and conform to well-established consensus sequences [24]. We also easily identified a variety of RNA *cis*-regulatory elements known as riboswitches [25] including two representatives of the glycine riboswitch class [26] previously described in '*Cand*. P. ubique' [27], two class II SAM riboswitches (SAM-II) [28] and a TPP riboswitch [29,30].

In contrast, identification of the tmRNA [31] representative was somewhat more challenging. The tmRNA eluded identification during initial screens for several reasons. First, in the genome of '*Cand*. P. ubique' the flanking gene (*thyX*, SAR11_0010) is likely misannotated resulting in a partial overlap of the annotated coding region with the tmRNA. While coding sequences in '*Cand*. P. ubique' often overlap by several nucleotides, an in-frame methionine at position 30 of the existing annotation for thymidylate synthase sequence is most likely the correct start site based on BLAST analysis of ThyX protein sequences. Second, the genomic sequence of the tmRNA is split and permuted relative to the mature form of the RNA in '*Cand*. P. ubique'. While this feature is shared by most other Alphaproteobacteria and by some Cyanobacteria [32], it makes identification of the RNA more difficult because the region between the two sections varies in length between 75 and 125 bp [33], and the permuted model is not currently represented in the Rfam database [24].

By applying length, %GC and conservation thresholds we have significantly enriched our list of IGRs for known structured RNAs. Only, 4% of all IGRs in '*Cand*. P. ubique' contain known structured RNAs. Approximately 17% of IGRs greater than 100 bp contain structured RNA; and eliminating IGRs with <26% GC increases this percentage to ~40%. Applying the BLAST hit threshold further increases percentage of considered IGRs containing known structured RNAs to ~75%. However, our parameter choices do exclude 2 of the 34 IGRs (6%) containing previously known RNAs. The first is a tRNA that is found within an IGR of 98 bp. We explored lowering the 100 bp threshold. However, we identified few additional candidates, and these candidates typically were very close to previously established thresholds for other parameters further decreasing their attractiveness for comprehensive study. The second example of a known RNA we excluded using our parameters is the IGR containing a SAM-II riboswitch preceding *metX *(SAR11_0217), which failed to rank highly based on GC-enrichment. The IGR containing this riboswitch is 191 nucleotides long and 22.5% GC (ranked 121^st ^in the genome based on Additional file 1). However, the SAM-II aptamer alone is 70 nucleotides long and 30% GC. An early investigation of the '*Cand*. P. ubique' genome did explore ranking the IGRs by the highest percent GC within a "sliding window" of 50 nucleotides [19]. However, this did not change the rankings of '*Cand*. P. ubique' IGRs significantly (R^2 ^= 0.84, Additional file 3). Thus, this additional level of complexity was not implemented for the final analysis.

For those IGRs that are longer than 100 bp, greater than 26% GC, and well-conserved in the marine metagenome (Table 1) but do not contain known structured RNAs, similar sequences identified by the BLAST analysis were used as input for comparative sequence analysis algorithms employed for ncRNA discovery. For each IGR several hypothetical alignments and secondary structures were generated using a covariance model search [34]. These alignments and predicted secondary structures were then used as the starting point for homology searches of the NCBI and metagenomic sequence databases to identify additional examples [35,36]. To confirm and refine secondary-structure models and sequence alignments, all examples for a particular IGR were subsequently combined and the process repeated beginning with the covariance model search to generate an RNA secondary structure that is well-supported by a large number of representatives (100–300 unique sequences).

**Figure 2:**
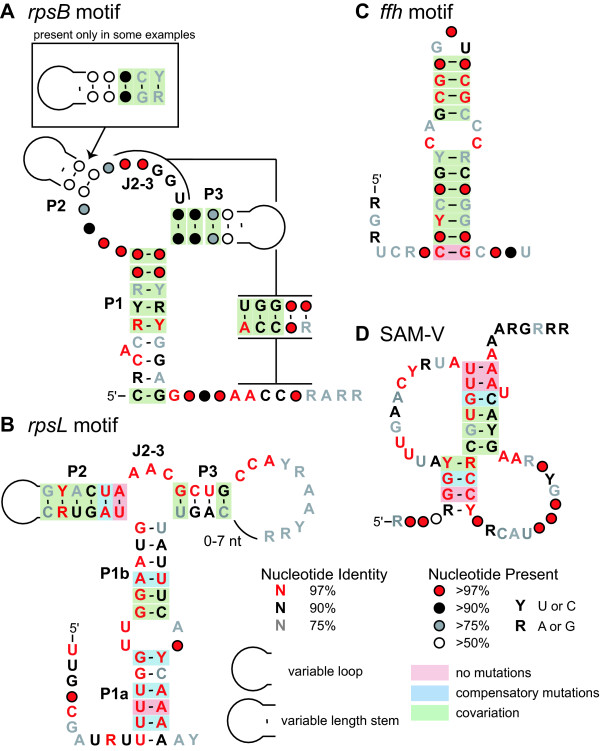
**Consensus sequences and structures for the four RNA motifs identified**. (A) *rpsB *motif, (B) *rpsL *motif, (C) *ffh *motif, (D) SAM-V riboswitch. See Additional files 4, 5, 6, 7 for alignments of all representatives. Calculations for conservation of nucleotide identity are described in the Methods section. Proposed base pairs with more than 5% non-canonical Watson-Crick pairings or missing nucleotides are not classified as covarying.

**Figure 3:**
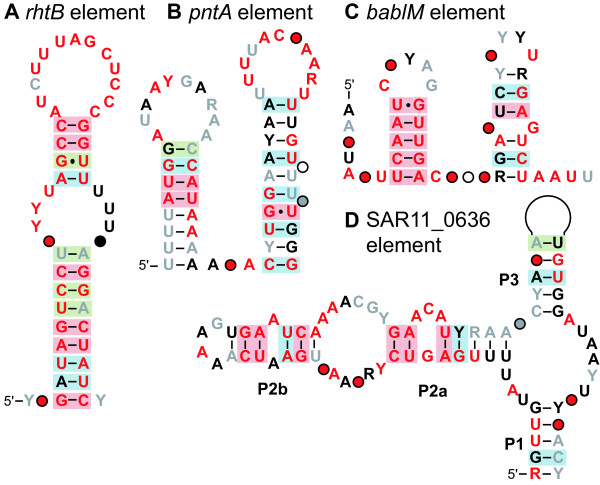
**The conserved sequence and secondary structure of the four candidate RNA motifs identified**. (A) *rhtB *associated element, (B) *pntA *associated element, (C) *bablM *associated element, (D) SAR11_0636 element. See Additional files 8, 9, 10, 11 for alignments of all representatives. Structural notations are as in Fig. 2, and consensus nucleotides and covariation computed identically to Fig. 2.

Using this strategy, we discovered candidate structured RNA elements located 5' relative to genes encoding ribosomal proteins S2 (*rpsB*) and S12 (*rpsL*), and the signal recognition particle protein (*ffh*). We also found a structured RNA element associated with genes for the methionine biosynthesis proteins *O*-acetylhomoserine (thiol)-lyase (*metY*), homoserine *S*-methyltransferase (*mmum*) and betaine-homocysteine methyltransferase (*bhmt*) (Figure 2). Moreover, we identified a series of IGRs that contain potential RNA structures that are less well-supported by the alignments and often include highly conserved regions with few mutations and thus few opportunities to observe covariation and compatible mutations that are the hallmark of a correctly predicted RNA secondary structure (Figure 3). Features of these new-found candidate structured RNAs are described below.

### *rpsB *motif

We identified a likely RNA motif preceding the gene *rpsB*, which encodes ribosomal protein S2. The motif is present in both marine metagenomic sequences and most Alphaproteobacteria with the exception of most members of the Rickettsiaceae family (Additional file 4). In addition, we identified representatives in most Gammaproteobacteria, a few Epsilon-, Delta-, and Betaproteobacteria, Cyanobacteria, and some Firmicutes. In nearly all examples where the downstream genes can be determined, the motif precedes *rpsB*. However, a few precede *fts*, which encodes elongation factor Ts (Ef-Ts) and is often found in the same operon as *rpsB *[37].

The structure of *rpsB *motif (Figure 2A) consists of a long base-paired structure (P1) capped by a three-stem junction carrying two variable length stems (P2 and P3), both of which may be very short, or absent in some representatives. The nucleotide junction between P2 and P3 (J2–3) forms a pseudoknot with the 3' extension following P1. P2 is quite short in '*Cand*. P. ubique' and consists of only three base pairs. In Cyanobacteria, Firmicutes, and most Gammaproteobacteria this pairing element is entirely absent or very short (three or fewer base pairs). In contrast, P2 is up to eleven base-pairs in some species of Alphaproteobacteria. P3 is also quite short in '*Cand*. P. ubique' with only two base pairs, however, it is typically at least four base pairs and has greater than twelve base pairs in several species of Alpha- and Gammaproteobacteria. The pseudoknot interaction is present across all of the taxa. However, in Firmicutes it appears to only consist of three base pairs rather than the five predicted in other phylogenetic groups.

*Cis*-regulatory elements in the 5' untranslated regions (UTRs) of ribosomal protein encoding mRNAs have long been known [38]. Ribosomal proteins L1 [39], L4 [40,41], L10/L12 [42], L20 [43], S4 [44,45], S7 [46], S8 [47,48], S15 [49], and S1 [50] are known to bind mRNA sequences to control gene expression. All such sequences characterized to date are autoregulatory, where the mRNA is bound by a ribosomal protein encoded within the transcript [38]. Typically such sequences inhibit translation, although some regulate transcription [41,51].

The role of the S2 ribosomal protein in translation is not well understood. S2 binds the 30S subunit late in ribosome biogenesis and acts as a bridge between the 16S RNA and ribosomal protein S1, which is the only ribosomal protein contacting the 30S subunit through protein-protein interactions [52]. The function of S1 is similarly unclear; however it has been implicated in translating highly structured mRNAs [53], as well as in the formation of the translation initiation complex at internal ribosome binding sites [54]. Analysis of the crystal structure of the 30S subunit from *T. Thermophilus *ribosome shows that S2 contacts distal regions of the 16S RNA (H26 in the body and H35–37 in the body) [55]. These regions bear no obvious resemblance to the motif we have identified. However, structural mimicry cannot be excluded. In several instances the 5' UTR of an mRNA and the ribosomal RNA bound by the same protein share similar tertiary structures despite having little or no primary or secondary structure similarity [56-59].

The region upstream of the ribosomal protein S2 was identified as a potential 5' UTR in a transcriptome analysis of *Escherichia coli *[60,61]. In addition, recent *in vivo *work in *E. coli *shows that the region 162 nucleotides upstream of *rpsB *controls an *rpsB-lacZ *fusion construct in response to exogenous S2 added in *trans *[62]. This work identified the conserved RNA structure upstream of *rpsB *in other Gammaproteobacteria. However, we identified a more broadly conserved motif in Alpha- Beta- and Deltaproteobacteria as well as Cyanobacteria and Firmicutes. In addition, the pseudoknot interaction had not previously been identified.

### *rpsL *motif

A second putative motif in the 5' UTR of a ribosomal mRNA was identified for *rpsL *(encoding ribosomal protein S12), the first gene in a series of 22 genes encoding ribosomal proteins in '*Cand. P. ubique*' that are homologous to those in the *E. coli str, spc*, and *S10 *ribosomal operons. We identified over 900 representatives (659 unique sequences) of the motif in the marine metagenome in addition to the instance in '*Cand*. P. ubique' (Additional file 5). The motif is consistently identified 3' of *rpoC*, which encodes RNA polymerase, and 5' of *rpsL*. The genes further downstream of *rpsL *are typically those identified in the '*Cand. P*. ubique' operon. However, due to the length of the metagenomic sequences analyzed it is impossible to determine whether the entire series of ORFs is conserved. The motif occasionally precedes *rpsG *or *fusA *genes that directly follow *rpsL *in the '*Cand*. P. ubique' genome. Despite extensive searching, we only identified the motif in '*Cand*. P. ubique' and marine metagenomic sequence samples.

The motif consists of a bulged P1 stem connecting to a three-stem junction (Figure 2B). The P2 stem shows covariation throughout its length, however, the loop region is diverse both in length (3–10 nt) and sequence. Both the P1 and P3 stems show some covariation, but more positions exhibit breaks in the Watson-Crick base pairing compared with the P2 stem. The nucleotides in J2–3 are identical in nearly all examples, and the P3 loop and P1 bulge also show extensive conservation.

Several proteins encoded by this series of ribosomal protein genes in '*Cand*. P. ubique' have been shown to regulate ribosomal protein expression in *E. coli *[40,41,46-48,62]. The *str *ribosomal operon (encoding ribosomal proteins S12, S7, and elongation factors G and Tu) is regulated by the binding of S7 to the transcript region between the genes for S12 and S7 [46]. Similarly, the *spc *operon (encoding ribosomal proteins L14, L24, L5, S14, S8, L6, L18, S5, L30 L15 and *secY*) is regulated by S8 binding to an mRNA structure between L24 and L5 [47,48]. The eleven-gene *S10 *operon (encoding ribosomal proteins S10, L3, L4, L23, L2, S19, L22, S3, L16, L29, S17) is regulated by ribosomal protein L4 binding to a 5' UTR preceding the S10 gene [40,41].

The secondary structure of the motif described here does not bear any resemblance to the regulatory motifs associated with S7, S8 and L4. Additionally, the *rpsL *motif is not located at the same genomic position as any of the *E. coli *regulatory motifs. While this series of ribosomal proteins in '*Cand*. P. ubique' essentially consists of the three separate *E. coli *operons, separate regulation in this organism is unlikely as the coding regions typically overlap by a few base pairs and the largest IGR is nine nucleotides. This motif is not identified outside of '*Cand*. P. ubique' and the metagenomic data. However, given its genomic context and conserved secondary structure, the *rpsL *motif is likely a structured RNA involved with regulation of ribosomal protein expression. Considering the large number of potential candidates, we cannot predict with confidence which protein may be its binding partner.

### *ffh *motif

We identified an RNA motif in the IGR preceding the gene *ffh *which encodes the cytoplasmic protein component of the bacterial signal recognition particle (SRP). The motif is well-conserved in metagenomic sequence samples with over 600 representatives (345 unique sequences) (Additional file 6). In addition, this motif is widespread among Alphaproteobacteria occurring in all fully-sequenced representatives of the Rhodobacterales, Sphingomonadales and Rhizobiales classes. However, the *ffh *motif does not occur in any sequenced representatives of the Rhodospirillales or Caulobacterales classes and it is also not found in representatives of Rickettsiales other than '*Cand*. P. ubique'. In nearly all examples where the downstream genes can be identified, the motif precedes *ffh*. This transcript has been detected by several metatranscriptomics analyses of microbial small RNAs [63,64].

The RNA motif consists of a single bulged hairpin (Figure 2C). However, there is convincing co-variation found at all positions along the stem with the exception of the first base-pair which is always a cytosine-guanosine pair. Additionally, there is significant sequence conservation within the bulge. In particular the two cytosine residues are found in nearly every example.

The signal recognition particle (SRP) is an essential RNA-protein complex conserved in all three domains of life that targets secreted proteins to the plasma membrane in eubacteria and archaea or to the endoplasmic reticulum in eukaryotes through interactions with peptide signal sequences [21]. The eubacterial SRP complex consists of the 4.5S RNA, a cytoplasmic protein (Ffh), and a receptor protein (FstY) that targets the complex to the membrane. Ffh binds directly to a conserved portion of the 4.5S RNA known as helix 8 [65], and FstY in turn binds Ffh [66,67]. The eukaryotic and archaeal SRPs typically consist of larger RNAs and a greater number of proteins. However, the interactions between the RNA component and the cytoplasmic protein are conserved [68].

How the levels of the Ffh protein and the 4.5S RNA are regulated is not fully understood. In *E. coli *the 4.5S RNA is present in excess compared to Ffh [69], and it has been shown using both depletion studies [70] and examination of a temperature sensitive *ffh *mutant in *E. coli *[71] that Ffh is significantly stabilized by its interactions with the 4.5S RNA and is rapidly degraded when not bound to the RNA. However, no regulation at the transcriptional or translational level has been described. The RNA motif identified does not appear to resemble the portion of the 4.5S RNA bound by Ffh. However, it is possible that the motif plays a role in the regulation of the *ffh *gene, especially given the widespread distribution of this motif and the precedent for *cis*-regulatory mRNA elements associated with the genes of RNA binding proteins [72].

### Methionine biosynthesis associated motif

We identified a conserved RNA motif preceding the methionine biosynthesis genes *mmum, metY*, and *bhmt*. This conserved sequence was previously identified as a potential regulatory region in '*Cand*. P. ubique' as the three genes appear to be co-regulated from proteomic studies [73]. We found 690 representatives (505 unique sequences) in metagenomic sequences, most of which precede *metY *(Additional file 7). However, there are metagenomic examples that precede *bhmt*, *metH*, and *mmum*. In addition, there is a single example in the genome of *Psychroflexus torquis *ATCC 700755 (RefSeq accession NZ_AAPR0000000) also preceding *metY*.

The motif consists of a simple pseudoknotted structure that is typically within ten nucleotides of a start codon (Figure 2D). Both stems show covariation and many loop nucleotides are well-conserved. Based on the association of the motif with methionine biosynthesis genes, the coregulation of the three genes in '*Cand*. P. ubique' [73], and the prevalence of *S*-adenosylmethionine (SAM)-binding riboswitches [74], we hypothesized that the RNA was a SAM-binding riboswitch. *In vitro *biochemical characterization of the RNA has revealed that representatives of this RNA motif selectively bind SAM (M. Meyer, E. Poiata, and R. Breaker; unpublished data).

The RNA motif also displays some similarities to the previously described class II SAM riboswitches (SAM-II) that bind SAM and control sulfur metabolism genes in Alphaproteobacteria [28]. In particular the two RNA motifs share a similar overall pseudoknotted structure and many of the bases shown to contact the ligand in a crystal structure of the class II SAM riboswitch [75] have equivalent nucleotides in the new-found motif. Despite these similarities, the motif lacks the final 3' base-pairing element present in most SAM-II riboswitch representatives. Moreover, both paired regions in the new motif differ in length from those in the SAM-II consensus, and the loop regions outside those that bind the ligand in the SAM-II riboswitch are not well conserved. Such differences in the riboswitch aptamers for SAM-I and SAM-IV riboswitches cause representatives to be sorted into distinct collections when examined using bioinformatics search algorithms that identify common sequence and structural elements [76]. Likewise, the differences between SAM-II and the new-found motif also cause them to be sorted independently, suggesting that this is a new class of SAM-binding riboswitches that we have termed SAM-V.

### Other potential RNA motifs

In addition to the motifs that we identified that have strong support as structured RNAs based on their alignments and distribution, we also identified several potential RNA motifs that are less well-supported. These candidate RNA motifs have fewer positions with covariation or compatible mutations and are not identified outside the genome of '*Cand*. P. ubique' and metagenomic sequences. However, they do exhibit evidence of possible RNA structure formation and our models are supported by sequence alignments from the marine metagenome.

The first of these motifs consists of a single bulged hairpin (Figure 3A). Both portions of the stem are conserved, and show indications of covariation and compatible mutations at many positions. Both the loop and the bulge are also well-conserved. The alignment consists of ~1250 representatives (919 unique sequences) from the marine metagenome and '*Cand*. P. ubique' (Additional file 8). In '*Cand*. P. ubique' the motif is flanked by a hypothetical protein and *rhtB *(LysE type translocator). In the metagenomic sequence, this context is largely conserved. However, the motif also appears upstream of *proC *(pyrroline-5-carboxylate reductase), as well as other genes further downstream of *rhtB *in '*Cand*. P. ubique' such as *livM *and *livK *(components of putative branched amino acid transporters). Approximately 50% of examples of this motif, including the one in '*Cand*. P. ubique', are directly followed by a poly-uridine track of 6–9 nucleotides potentially forming a rho-independent terminator stem [77]. This feature suggests either a potential regulatory function or a conserved termination signal. However, the lower portion of the well-conserved hairpin structure also forms a fairly convincing inverted repeat sequence, which may indicate alternative functionality.

The second motif consists of two base-paired stems in series where the loop of the second is especially well-conserved (Figure 3B). The alignment includes 365 unique sequences derived from metagenomic sequences (~400 total representatives), in addition to the example in '*Cand*. P. ubique' (Additional file 9). In '*Cand*. P. ubique' the motif is flanked by *rpmJ*, which encodes the ribosomal protein L36, and *pntA*, which encodes the alpha subunit of a pyridine nucleotide transhydrogenase. In the marine metagenome the motif consistently precedes *pntA*, but the gene annotated directly 5' of the motif varies. Most frequently it is the 5S rRNA gene, or *rmlB *(dTDP-D-glucose 4,6-dehydratase, COG1088). The conserved position of this motif 5' of the *pntA *gene suggests a regulatory function related to *pntA*. However, there is an additional ~60 bp of sequence between the motif and the start of the gene. While this sequence is somewhat conserved at the nucleotide level, this region does not appear to have any structure supported by compatible or covarying base-pair interactions.

The third motif (Figure 3C) also consists of a set of predicted base-pairing stems in series. The sequence of the first predicted stem is very strongly conserved, with no mutations observed in any of the representatives identified. The second stem shows a few compatible mutations and the position nearest the loop frequently fails to maintain base pairing. The loops and linker regions exhibit almost no conservation. Approximately 540 representatives (314 unique sequences) were identified in the marine metagenome, and the genomic context is well conserved (Additional file 10). The motif occurs between *rnhB1 *(RNaseHII) and *bablM *(a site-specific DNA methylase) in the genome of '*Cand*. P. ubique' and the vast majority of metagenomic examples fall between genes annotated as *rnhB1 *and a DNA methylase.

The fourth motif is somewhat more complex than others in this category (Figure 3D). There are ~640 representatives (338 unique sequences) in the marine metagenome in addition to that in the genome of '*Cand*. P. ubique' (Additional file 11). Its three-stem junction carries a well conserved stem (P2) that contains two bulged regions, one of which is highly conserved. Due to this conservation, none of the base pairs are supported by covariation and only a few by compatible mutations. The other two stems (P1 and P3) are only moderately conserved, and the loop of P3 is variable in length containing between 5 and 12 nucleotides with no strong conservation. The motif occurs between two hypothetical proteins. One (SAR11_0635) is annotated as both an SOS-mediated transcriptional repressor and an S24-like peptidase depending on the database, and the other (SAR11_0636) is annotated as a SOUL heme-binding protein. In the metagenomic data, neither of these associations is strictly conserved and the annotated genes on either side vary widely. The genes annotated directly 5' to the motif are typically syntenous with those in '*Cand*. P. ubique' (i.e. predicted glycoyltransferase, SAR11_0633). The genes annotated directly 3' of the motif show even greater variation and do not seem to be syntenous with the '*Cand*. P. ubique' genome. Based on these observations, it seems likely that the RNA is not a *cis*-regulatory element, but rather could be a separately transcribed non-coding RNA.

Microarray studies show that transcripts for all of these genes, although not necessarily any untranslated regions, are present in '*Cand*. P. ubique' during both exponential growth and stationary phase cells. Interestingly, comparison of microarray and quantitative proteomic data (unpublished data) for *pntA *shows a ~300% increase in protein as cells enter stationary phase, starkly contrasting the corresponding 9% decease in transcript levels. This disparity between transcript and protein expression provides further evidence for post-transcriptional regulation of the gene. Unfortunately, proteomic data are not available for RhtB and BabIM (not included in the AMT-tag library), and SAR11_0636 was never observed in the proteomic dataset, so direct comparisons are not possible for these genes.

## Discussion

In this study we identified structured RNAs that are conserved in both the genome of '*Cand*. P. ubique' and the marine metagenomic datasets. A few these RNAs were assigned to previously-known classes, while this is the first description of others. Our work differs from other surveys of ncRNAs in the metagenome [6,7] in that we did not seek to identify additional examples of known motifs, but rather we sought to discover motifs not previously described. We identified three likely *cis*-regulatory protein binding motifs and a new riboswitch class, and our approach is validated by the confirmed biological function for two of the four motifs (*rpsB *motif and SAM-V riboswitch). In addition to these four RNA *cis*-regulatory elements, we also describe a series of motifs for which there is less evidence of RNA structure. While these RNA motifs are less well-supported by compatible and covarying mutations than the others we present, the structures are credible given the number of representatives identified, the degree of sequence conservation, and the thermodynamics of RNA folding.

There are many additional IGRs in '*Cand*. P. ubique' that contain a high percentage GC and seem highly conserved (Table 1), yet have no discernable RNA structure. For some of these IGRs, the large number of BLAST hits is the result of many different short aligned sections of high identity within the IGR (e.g. the IGR between SAR11_0641 and SAR11_0642). By contrast, in the IGRs where we identified convincing structured RNAs there is typically a longer region of alignment with mutations distributed throughout. For several other IGRs there are a large number of BLAST hits that align but form no detectable RNA structure (e.g. the IGR between SAR11_0037 and SAR11_0038). These regions may contain RNAs that are not extensively structured (e.g. antisense RNAs that base pair to target RNAs) [78], or perhaps they are conserved protein binding sites that act at the level of DNA.

The parameters we used to identify IGRs for inspection were based on the properties of previously annotated RNAs and were designed to capture most structured RNAs. However, one IGR containing a known structured RNA does not meet our parameters for inspection. The IGR containing a SAM-II riboswitch preceding *metX *(SAR11_0217) failed to rank highly based on GC-enrichment. The IGR containing this riboswitch is 191 nucleotides long and 22.5% GC (ranked 121^st ^in the genome based on Additional file 1), significantly below where we arbitrarily stopped examining IGRs due to the decreasing number of convincing BLAST matches (Table 1). However, the SAM-II aptamer alone is 70 nucleotides long and 30% GC. An early investigation of the '*Cand*. P. ubique' genome did explore ranking the IGRs by the highest percent GC within a "sliding window" of 50 nucleotides [19]. However, this did not change the rankings of '*Cand*. P. ubique' IGRs significantly (R^2 ^= 0.84, Additional file 11). Thus, this additional level of complexity was not implemented for the final analysis.

In contrast to other computational genomics studies [3], we identified relatively few candidate RNAs. This is likely because there is relatively little to find in '*Cand*. P. ubique' compared with organisms that have larger genomes. The genome of '*Cand*. P. ubique' is hypothesized to be streamlined to minimize nutrient use [14,79]. Even the strong AT bias may reflect adaptation to nitrogen limitation in a nutrient poor environment because GC pairs require an additional nitrogen compared to AT base pairs. A survey examining lengths of the RNase P RNA, SRP RNA, TPP and glycine riboswitches in '*Cand*. P. ubique' compared with those in other Alphaproteobacteria showed that RNAs in '*Cand*. P. ubique' have tendency toward fewer nucleotides (Additional file 12). On average they are greater than one standard deviation lower than the mean for a given RNA (average Z-value of -1.12). While this result is not statistically significant, the motifs identified here further reflect this tendency. The S2 motif identified in '*Cand*. P. ubique' is among the shortest with an exceedingly short P2 stem (3 bp) and no P3 stem. The presence of RNA-based regulatory motifs in '*Cand*. P. ubique' indicates that such mechanisms can be an effective use of scarce resources, and the smaller RNAs likely reflect pressure to decrease the number of nucleotides at both the DNA and RNA level. Interestingly ribosomal RNAs and tRNAs both showed less variation in length among Alphaproteobacteria than other structured RNAs, as well as less or no evidence of reduction in '*Cand*. P. ubique' suggesting that it is difficult to alter RNAs with functions critical for survival.

## Conclusion

This study increased the number of candidate structured RNAs in both '*Cand*. P. ubique' and the marine metagenome. Several of the RNAs discovered have wide phylogenetic distribution, while others can only be found through examination of metagenomic data. The combination of computational approaches used in this work is relatively simple and in principle might be applied to any organisms with similar properties. This work also underscores how single completed genomes that are carefully annotated are important components in the effort toward annotating and understanding the vast amount metagenomic data available.

## Methods

### Identification of candidate RNA motifs

Non-protein coding segments of the '*Cand*. P. ubique' genome (RefSeq accession number NC_007205.1) were computationally identified based on the RefSeq version 25 gene annotations and their sequences extracted [80]. The size and percent GC values for these regions were established. Individual sequences annotated as harboring a structured ncRNA according to the Rfam database (version 8.1) were identified [24]. Two additional sequences containing tRNAs were identified from the RefSeq annotation of the '*Cand*. P. ubique' genome, and the riboswitches were located based on alignments maintained through periodic homology searches [81].

As all known structured RNAs in '*Cand*. P. ubique' are present in IGRs longer than 100 bp (Fig. 1), we used 100 bp as the minimum size requirement for the IGRs we examined. The conservation level for each IGR was determined by the number of hits returned with an E-value less than 10^-5 ^from a nucleotide BLAST analysis of the IGR against the "GOS: All Metagenomic Sequence Reads" database maintained at the CAMERA website [20]. IGRs not well-conserved in metagenomic sequence data (less than 200 blast hits) were removed from consideration. The remaining IGRs were screened for the presence of unannotated protein coding regions first through BLASTX and subsequently TBLASTX searches of the NCBI nr and nr/nt databases  and TBLASTN searches of the "All Metagenomic Sequence Reads" CAMERA database. Those sequences containing a conserved protein coding region (Additional File 2) were excluded from further analysis.

For the remaining IGRs, all blast matches from the conservation analysis were collected and the sequences extended to match the length of the IGR, or to the end of the sequence read (average trimmed sequence read is 822 bp in length [13]). This collection of sequences was then used as input for CMFinder version 0.2 [34] which created multiple sequence alignments with putative conserved secondary structures. These alignments were manually examined for features indicative of a structured RNA such as extent of covariation within predicted stems and conservation in areas outside base-paired regions. For most IGRs, several alternative structures were initially chosen for further analysis due to the high level of conservation in the sequences.

The alignments and hypothetical secondary structures were used to search for additional homologs in the RefSeq25 database [80] along with metagenome sequences from acid mine drainage [82], soil and whale fall [83], human gut [84,85], mouse gut [86], gutless sea worms [87], sludge [88], Global Ocean Survey scaffolds [12,13], other marine sequences [89] and termite hindgut [90].

Homology searches were performed using RAVENNA version 0.2f, essentially as described previously [35,36,91,92]. For each IGR, homologs resulting from these searches were used in conjunction with the original sequences as the starting input for a second CMFinder search and the homology search process was iterated to derive a single structure, or in cases of predicted pseudoknot interactions two compatible structures, supported by the alignment.

### Analysis of motifs

The alignments of IGRs where convincing RNA structure could be identified were manually edited by RALEE [93]. We used RNAshapes [94], CMFinder [34] and RAVENNA [36] during these analyses. Additional homology searches were conducted using the RAVENNA '-local' and '-global' command line options with the microbial subset of RefSeq version 25, and the metagenomic sequence databases described above. As the full RefSeq database is 3,717,469,431 nucleotides and the combined metagenomic databases total 5,529,658,033 nucleotides, several subset databases (Proteobacteria, Alphaproteobacteria, Bacteroidetes, Additional File 2 and Global Ocean Survey Scaffolds) were used to reduce the number of false positive hits. Local searches tended to have greater success identifying homologs of motifs with variable length or optional stems.

For the genome context annotations, protein-coding genes were assembled from the annotations in RefSeq and from "predicted proteins" [5] in Global Ocean Survey sequences or annonatated genes in IMG/M [95]. However, sequences from three metagenome projects [85,89,90] were extracted from GenBank and genes were predicted using the MetaGene program (dated Oct. 12, 2006) with default parameters [96]. Conserved protein domains were detected using the Conserved Domain Database version 2.08 [97].

The extent of covariation and conservation of sequences reflected in consensus diagrams (e.g. Figure 2) was determined as previously described [92]. Sequences were weighted to de-emphasize highly similar homologs using the GSC algorithm [98] implemented by Infernal [35]. Base pairs where both positions in the sequence alignment varied among sequences while maintaining Watson-Crick or G-U wobble base pairing were classified as covarying. Base pairs where a single position varied were classified as compatible mutations. If the frequency of non-Watson-Crick or G-U pairs exceeded 5%, no covariation or compatible mutation was annotated.

## List of abbreviations

IGR: intergenic region; ncRNA: noncoding RNA; GOS: Global Oceanographic Survey; CAMERA: Community Cyberinfrastructure for Advanced Marine Microbial Ecology Research and Analysis; SRP: signal recognition particle; UTR: untranslated region; SAM: *S*-adenosylmethionine; bp: base pair.

## Authors' contributions

MMM conceived and designed the study, executed bioinformatics searches, analyzed the data, and drafted the manuscript. TDA participated in the design of the study and provided bioinformatics infrastructure. DPS conceived the study, performed proteomics searches, and revised the manuscript. ZW provided bioinformatics infrastructure and reviewed motif analysis. MSS conceived the study and revised the manuscript. SJG conceived the study and revised the manuscript. RRB participated in the design of the study, reviewed motif analysis, and revised the manuscript. All authors read and approved the final manuscript.

## Supplementary Material

Additional file 1 **All '*Cand*. P. ubique' IGRs greater than 100 bp**. A list of all intergenic regions in '*Cand*. P. ubique' longer than 100 bp with the length, GC content and annotated RNAs indicated. Click here for file

Additional file 2 **Misannotated protein coding regions identified**. A list of likely misannotated protein coding regions identified in the course of this study. Click here for file

Additional file 3 **IGR ranking by %GC and sliding window %GC**. Comparison of ranking IGRs by %GC and an alternative ranking methodology based on a sliding window of 50 nucleotides. Click here for file

Additional file 4 **rpsB alignment**. Text file containing Stockholm alignment of the *rpsB *motif, may be viewed in any text editor including XEmacs with the RALEE extension, or MS-wordpad. Click here for file

Additional file 5 **rpsL alignment**. Text file containing Stockholm alignment of the *rpsL *motif, may be viewed in any text editor including XEmacs with the RALEE extension, or MS-wordpad. Click here for file

Additional file 6 **ffh alignment**. Text file containing Stockholm alignment of the *ffh *motif, may be viewed in any text editor including XEmacs with the RALEE extension, or MS-wordpad. Click here for file

Additional file 7 **SAMV alignment**. Text file containing Stockholm alignment of the SAM-V motif, may be viewed in any text editor including XEmacs with the RALEE extension, or MS-wordpad. Click here for file

Additional file 8 **rhtb alignment**. Text file containing Stockholm alignment of the *rhtb *motif, may be viewed in any text editor including XEmacs with the RALEE extension, or MS-wordpad. Click here for file

Additional file 9 **pntA alignment**. Text file containing Stockholm alignment of the *pntA *motif, may be viewed in any text editor including XEmacs with the RALEE extension, or MS-wordpad. Click here for file

Additional file 10 **bablM alignment**. Text file containing Stockholm alignment of the *bablM *motif, may be viewed in any text editor including XEmacs with the RALEE extension, or MS-wordpad. Click here for file

Additional file 11 **SAR11_0636 alignment**. Text file containing Stockholm alignment of the SAR11_0636 motif, may be viewed in any text editor including XEmacs with the RALEE extension, or MS-wordpad. Click here for file

Additional file 12 **RNA motifs from Alphaproteobacteria ordered by length**. Glycine riboswitch, TPP riboswitch, SRP, and RNaseP RNAs from Alphaproteobacteria ordered by length. Click here for file
