# Supplementary material for: Identification of candidate structured RNAs in the marine organism 'Candidatus Pelagibacter ubique'
Source: BMC Genomics. 2009 Jun 16;10:268. doi: 10.1186/1471-2164-10-268 (PMC2704228; doi:10.1186/1471-2164-10-268)
Supplement: Additional file 1 — All 'Cand. P. ubique' IGRs greater than 100 bp. A list of all intergenic regions in 'Cand. P. ubique' longer than 100 bp with the length, GC content and annotated RNAs indicated. [file 1471-2164-10-268-S1.doc]

All ‘*Cand.* P. ubique’ IGRs longer than 100 bp ranked by % GC

| **Coordinates** | | | **Length** | **%GC** | **Annotated RNA(s)** |
| --- | --- | --- | --- | --- | --- |
| 568414 | - | 568514 | 101 | 51.49 | tRNA-Met, tRNA-Ile |
| 10302 | - | 10518 | 217 | 48.85 | tmRNA* |
| 1085414 | - | 1085515 | 102 | 48.04 | tRNA-Trp |
| 1087835 | - | 1087947 | 113 | 45.13 | tRNA-Thr |
| 594377 | - | 594492 | 116 | 43.97 | tRNA-Arg |
| 511028 | - | 516441 | 5414 | 43.31 | 23S ribosomal RNA, 16S ribosomal RNA, tRNA-Ala |
| 849107 | - | 849409 | 303 | 42.90 | tRNA-Asp, tRNA-Val |
| 990217 | - | 990346 | 130 | 42.31 | tRNA-Glu |
| 986128 | - | 986301 | 174 | 41.95 | tRNA-Pro |
| 649763 | - | 649953 | 191 | 41.88 | glycine riboswitch |
| 1031753 | - | 1031912 | 160 | 41.25 | tRNA-Ser |
| 564480 | - | 564659 | 180 | 40.00 | 5S ribosomal RNA |
| 41654 | - | 41899 | 246 | 39.84 | tRNA-Val, tRNA-Met |
| 116148 | - | 116256 | 109 | 39.45 | tRNA-Gln |
| 1086707 | - | 1087090 | 384 | 37.76 | tRNA-Tyr, tRNA-Gly |
| 493521 | - | 493664 | 144 | 36.81 | 4.5 S RNA* |
| 1127293 | - | 1127553 | 261 | 36.78 |  |
| 1088728 | - | 1088895 | 168 | 36.31 | tRNA-Leu |
| 1166455 | - | 1166736 | 282 | 35.82 | tRNA-Met |
| 564786 | - | 564910 | 125 | 35.20 |  |
| 466551 | - | 466719 | 169 | 34.91 | tRNA-Phe |
| 975329 | - | 975510 | 182 | 34.62 | tRNA-Ser |
| 38796 | - | 39447 | 652 | 34.51 | Rnase P RNA* |
| 415408 | - | 415585 | 178 | 34.27 | tRNA-Arg |
| 260190 | - | 260348 | 159 | 33.96 |  |
| 942390 | - | 942738 | 349 | 33.81 | tRNA-Cys, tRNA-Asn |
| 626974 | - | 627168 | 195 | 33.33 |  |
| 786467 | - | 786574 | 108 | 33.33 | TPP riboswitch* |
| 585015 | - | 585135 | 121 | 33.06 |  |
| 299224 | - | 299341 | 118 | 33.05 |  |
| 498458 | - | 498706 | 249 | 32.93 | glycine riboswitch |
| 622388 | - | 622552 | 165 | 32.73 |  |
| 319549 | - | 319742 | 194 | 32.47 | tRNA-Gly |
| 1142870 | - | 1143031 | 162 | 32.10 |  |
| 159067 | - | 159166 | 100 | 32.00 |  |
| 1292813 | - | 1292925 | 113 | 31.86 |  |
| 1120412 | - | 1120856 | 445 | 31.46 |  |
| 971264 | - | 971423 | 160 | 31.25 | tRNA-Lys |
| 843024 | - | 843225 | 202 | 31.19 | tRNA-Leu |
| 777150 | - | 777303 | 154 | 31.17 |  |
| 873155 | - | 873283 | 129 | 31.01 |  |
| 461376 | - | 461642 | 267 | 30.71 |  |
| 274773 | - | 275136 | 364 | 30.49 |  |
| 466933 | - | 467247 | 315 | 30.48 | tRNA-His |
| 628285 | - | 628539 | 255 | 30.20 |  |
| 1005679 | - | 1005890 | 212 | 30.19 |  |
| 361353 | - | 361571 | 219 | 30.14 |  |
| 1 | - | 515 | 515 | 30.10 |  |
| 1125490 | - | 1125606 | 117 | 29.91 |  |
| 400250 | - | 400460 | 211 | 29.86 |  |
| 936064 | - | 936341 | 278 | 29.86 | tRNA-Arg |
| 1163072 | - | 1163668 | 597 | 29.82 | tRNA-Met |
| 1189853 | - | 1189956 | 104 | 29.81 |  |
| 676100 | - | 676378 | 279 | 29.75 |  |
| 1212757 | - | 1212865 | 109 | 29.36 |  |
| 1277156 | - | 1277496 | 341 | 29.33 |  |
| 732778 | - | 732938 | 161 | 29.19 |  |
| 799165 | - | 799421 | 257 | 29.18 |  |
| 57720 | - | 58035 | 316 | 29.11 |  |
| 120095 | - | 120215 | 121 | 28.93 |  |
| 106370 | - | 106799 | 430 | 28.60 |  |
| 762114 | - | 762332 | 219 | 28.31 |  |
| 462363 | - | 462539 | 177 | 28.25 | tRNA-Thr |
| 834435 | - | 834636 | 202 | 28.22 |  |
| 1E+06 | - | 1164384 | 146 | 28.08 |  |
| 408449 | - | 408797 | 349 | 28.08 | tRNA-Leu |
| 52729 | - | 52885 | 157 | 28.03 |  |
| 1297623 | - | 1297755 | 133 | 27.82 |  |
| 675041 | - | 675166 | 126 | 27.78 |  |
| 762678 | - | 763012 | 335 | 27.76 |  |
| 43688 | - | 43789 | 102 | 27.45 |  |
| 791867 | - | 792012 | 146 | 27.40 |  |
| 1132812 | - | 1132928 | 117 | 27.35 |  |
| 1123617 | - | 1123934 | 318 | 27.04 |  |
| 1181972 | - | 1182071 | 100 | 27.00 |  |
| 670506 | - | 670772 | 267 | 26.97 |  |
| 1E+06 | - | 1074359 | 171 | 26.90 |  |
| 164139 | - | 164261 | 123 | 26.83 |  |
| 1245732 | - | 1245856 | 125 | 26.40 |  |
| 483528 | - | 483671 | 144 | 26.39 |  |
| 1201243 | - | 1201342 | 100 | 26.00 |  |
| 666745 | - | 666876 | 132 | 25.76 |  |
| 64904 | - | 65035 | 132 | 25.76 |  |
| 1117444 | - | 1117611 | 168 | 25.60 |  |
| 1203423 | - | 1203571 | 149 | 25.50 |  |
| 1164787 | - | 1164990 | 204 | 25.49 |  |
| 1119219 | - | 1119466 | 248 | 25.40 |  |
| 84917 | - | 85063 | 147 | 25.17 |  |
| 625718 | - | 625896 | 179 | 25.14 |  |
| 1255817 | - | 1256000 | 184 | 25.00 |  |
| 339536 | - | 339852 | 317 | 24.61 |  |
| 396319 | - | 396518 | 200 | 24.50 |  |
| 1236319 | - | 1236433 | 115 | 24.35 |  |
| 1136147 | - | 1136253 | 107 | 24.30 |  |
| 80106 | - | 80344 | 239 | 24.27 |  |
| 804600 | - | 804703 | 104 | 24.04 |  |
| 414757 | - | 414885 | 129 | 24.03 |  |
| 53027 | - | 53176 | 150 | 24.00 |  |
| 1037815 | - | 1037914 | 100 | 24.00 |  |
| 550670 | - | 550865 | 196 | 23.98 |  |
| 89735 | - | 89885 | 151 | 23.84 |  |
| 184247 | - | 184377 | 131 | 23.66 |  |
| 502373 | - | 502575 | 203 | 23.65 |  |
| 232153 | - | 232262 | 110 | 23.64 |  |
| 410316 | - | 410671 | 356 | 23.60 |  |
| 291667 | - | 291789 | 123 | 23.58 |  |
| 618241 | - | 618355 | 115 | 23.48 |  |
| 312736 | - | 312872 | 137 | 23.36 |  |
| 268313 | - | 268432 | 120 | 23.33 |  |
| 59846 | - | 60068 | 223 | 23.32 |  |
| 948566 | - | 948677 | 112 | 23.21 |  |
| 1273961 | - | 1274094 | 134 | 23.13 |  |
| 289493 | - | 289674 | 182 | 23.08 |  |
| 559030 | - | 559269 | 240 | 22.92 |  |
| 791451 | - | 791590 | 140 | 22.86 |  |
| 999830 | - | 1000425 | 596 | 22.82 |  |
| 722424 | - | 722559 | 136 | 22.79 |  |
| 542855 | - | 543012 | 158 | 22.78 |  |
| 770684 | - | 770784 | 101 | 22.77 |  |
| 1171740 | - | 1171934 | 195 | 22.56 |  |
| 218143 | - | 218333 | 191 | 22.51 | SAM-II riboswitch |
| 1153704 | - | 1153903 | 200 | 22.50 |  |
| 1306091 | - | 1306296 | 206 | 22.33 |  |
| 205724 | - | 205871 | 148 | 22.30 |  |
| 470365 | - | 470545 | 181 | 22.10 |  |
| 398434 | - | 398914 | 481 | 22.04 |  |
| 46027 | - | 46154 | 128 | 21.88 |  |
| 1210797 | - | 1210948 | 152 | 21.71 |  |
| 735071 | - | 735367 | 297 | 21.55 |  |
| 833166 | - | 833342 | 177 | 21.47 |  |
| 893454 | - | 893556 | 103 | 21.36 |  |
| 716080 | - | 716319 | 240 | 21.25 |  |
| 342918 | - | 343082 | 165 | 21.21 |  |
| 422877 | - | 423060 | 184 | 21.20 |  |
| 168181 | - | 168375 | 195 | 21.03 |  |
| 703381 | - | 703604 | 224 | 20.98 |  |
| 175289 | - | 175398 | 110 | 20.91 |  |
| 783218 | - | 783434 | 217 | 20.74 |  |
| 972201 | - | 972374 | 174 | 20.69 |  |
| 940612 | - | 940785 | 174 | 20.69 |  |
| 839683 | - | 839934 | 252 | 20.63 |  |
| 601228 | - | 601339 | 112 | 20.54 |  |
| 998829 | - | 999126 | 298 | 20.47 |  |
| 614086 | - | 614301 | 216 | 20.37 |  |
| 767476 | - | 767593 | 118 | 20.34 |  |
| 351819 | - | 352027 | 209 | 20.10 |  |
| 416774 | - | 416903 | 130 | 20.00 |  |
| 1115568 | - | 1115723 | 156 | 19.87 |  |
| 730367 | - | 730512 | 146 | 19.86 |  |
| 837367 | - | 837473 | 107 | 19.63 |  |
| 1209656 | - | 1209773 | 118 | 19.49 |  |
| 1175694 | - | 1175832 | 139 | 19.42 |  |
| 1207515 | - | 1207628 | 114 | 19.30 |  |
| 623486 | - | 623594 | 109 | 19.27 |  |
| 641772 | - | 641906 | 135 | 19.26 |  |
| 389597 | - | 389700 | 104 | 19.23 |  |
| 75771 | - | 77114 | 1344 | 18.97 |  |
| 488276 | - | 488407 | 132 | 18.94 |  |
| 812076 | - | 812186 | 111 | 18.92 |  |
| 823942 | - | 824060 | 119 | 18.49 |  |
| 555053 | - | 555223 | 171 | 18.13 |  |
| 430507 | - | 430650 | 144 | 18.06 |  |
| 335453 | - | 335796 | 344 | 17.73 |  |
| 1225927 | - | 1226063 | 137 | 17.52 |  |
| 491845 | - | 491947 | 103 | 17.48 |  |
| 1159707 | - | 1159818 | 112 | 16.96 |  |
| 921866 | - | 921990 | 125 | 16.80 |  |
| 790178 | - | 790316 | 139 | 16.55 |  |
| 1183889 | - | 1183999 | 111 | 16.22 |  |
| 62205 | - | 62322 | 118 | 16.10 |  |
| 561445 | - | 561626 | 182 | 15.93 |  |
| 66332 | - | 66489 | 158 | 15.82 |  |
| 487604 | - | 487774 | 171 | 15.79 |  |
| 707740 | - | 707854 | 115 | 14.78 |  |
| 441082 | - | 441233 | 152 | 14.47 |  |
| 1218828 | - | 1218953 | 126 | 14.29 |  |
| 1098498 | - | 1098632 | 135 | 14.07 |  |
| 1133946 | - | 1134060 | 115 | 13.91 |  |
| 713644 | - | 713745 | 102 | 13.73 |  |
| 829826 | - | 829949 | 124 | 12.90 |  |
| 745524 | - | 745723 | 200 | 12.00 |  |

*Annotated by this study.
