# Supplementary material for: Identification of candidate structured RNAs in the marine organism 'Candidatus Pelagibacter ubique'
Source: BMC Genomics. 2009 Jun 16;10:268. doi: 10.1186/1471-2164-10-268 (PMC2704228; doi:10.1186/1471-2164-10-268)
Supplement: Additional file 2 — Misannotated protein coding regions identified. A list of likely misannotated protein coding regions identified in the course of this study. [file 1471-2164-10-268-S2.doc]

Supplemental Table 2: Likely misannotated protein coding regions identified in the course of this study.

| **Coordinates** | |  | **Length** | **%GC** | **Blast Hits < 1 x 10 -5** | **Comments on Protein Sequence** |
| --- | --- | --- | --- | --- | --- | --- |
| 777150 | - | 777303 | 154 | 31.17 | 184 | in frame atg at 777254 likely *pta* start |
| 461376 | - | 461642 | 267 | 30.71 | 429 | *rpmH* ribosomal protein L34 (461550-461409) |
| 274773 | - | 275136 | 364 | 30.49 | 569 | in frame atg at 274976 likely *adhP* start |
| 400250 | - | 400460 | 211 | 29.86 | 889 | in frame atg at 400239 likely *dnaA* start |
| 676100 | - | 676378 | 279 | 29.75 | 1074 | contains peptide (676368-676308) showing similarity to N terminus of PU1002_02921. |
| 1277156 | - | 1277496 | 341 | 29.33 | 1069 | in frame atg at 1277323 is likely monooxygenase start |
| 799165 | - | 799421 | 257 | 29.18 | 510 | in frame atg at 799350 is likely *metC* start |
| 106370 | - | 106799 | 430 | 28.60 | 159 | likely pseudogene, long uninterrupted ORF with no apparent start, similar to hypothetical proteins in other alpha- proteobactera |
| 299224 | - | 299341 | 118 | 33.05 | 539 | contains portion of conserved protein |
