# Supplementary material for: Identification of candidate structured RNAs in the marine organism 'Candidatus Pelagibacter ubique'
Source: BMC Genomics. 2009 Jun 16;10:268. doi: 10.1186/1471-2164-10-268 (PMC2704228; doi:10.1186/1471-2164-10-268)
Supplement: Additional file 3 — IGR ranking by %GC and sliding window %GC. Comparison of ranking IGRs by %GC and an alternative ranking methodology based on a sliding window of 50 nucleotides. [file 1471-2164-10-268-S3.doc]

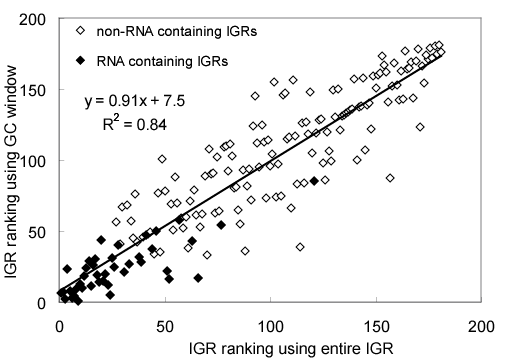


**Supplementary Figure 1:** ‘*Cand.* P. ubique’ IGRs longer than 100 bp were ranked by the percent GC over the entire length of the predicted IGR and by the highest percent GC calculated for a 50 bp window within the IGR. While there are differences between the two rankings they were not considered significant enough to alter the analysis method.
