# Supplementary material for: Identification of candidate structured RNAs in the marine organism 'Candidatus Pelagibacter ubique'
Source: BMC Genomics. 2009 Jun 16;10:268. doi: 10.1186/1471-2164-10-268 (PMC2704228; doi:10.1186/1471-2164-10-268)
Supplement: Additional file 12 — RNA motifs from Alphaproteobacteria ordered by length. Glycine riboswitch, TPP riboswitch, SRP, and RNaseP RNAs from Alphaproteobacteria ordered by length. [file 1471-2164-10-268-S12.doc]

Supplemental Table 3: RNA motifs from Alphaproteobacteria ordered by length. ‘*Cand.* P. ubique’ sequences are bolded.

| **Glycine Riboswitch** |  | **TPP riboswitch** |  |
| --- | --- | --- | --- |
| **RefSeq Accession** | **Length** | **RefSeq Accession** | **Length** |
| NZ_AANB01000012.1/35681-35540 | 142 | **NC_007205.1/786499-786580** | **82** |
| **NC_007205.1/498615-498458** | **158** | NC_008209.1/1392877-1392958 | 82 |
| NZ_AATR01000001.1/97815-97974 | 160 | NC_008209.1/3473673-3473754 | 82 |
| NZ_AAYB01000002.1/662791-662952 | 162 | NZ_AAYC01000017.1/65404-65320 | 85 |
| NC_003047.1/1674991-1674829 | 163 | NZ_AAYA01000014.1/50163-50250 | 88 |
| NC_009428.1/2340326-2340164 | 163 | NC_007765.1/373229-373140 | 90 |
| NC_009636.1/1265496-1265334 | 163 | NC_008384.1/477354-477265 | 90 |
| NZ_AALV01000008.1/136702-136864 | 163 | NZ_AANB01000002.1/179205-179294 | 90 |
| NZ_AALZ01000002.1/435131-435293 | 163 | NZ_AAPJ01000005.1/279112-279023 | 90 |
| NZ_AAXZ01000002.1/235345-235507 | 163 | NC_007493.1/2388684-2388594 | 91 |
| NZ_AAYA01000005.1/56287-56449 | 163 | NC_009049.1/2435527-2435437 | 91 |
| NZ_AAMO01000011.1/102939-103102 | 164 | NC_009428.1/584969-585059 | 91 |
| NZ_AATQ01000026.1/62712-62875 | 164 | NC_009720.1/2483104-2483194 | 91 |
| NZ_ABCR01000010.1/40880-41043 | 164 | NC_008358.1/3488012-3488103 | 92 |
| NC_007493.1/804595-804759 | 165 | NZ_AAMO01000004.1/229677-229586 | 92 |
| NZ_AAMV01000018.1/15473-15637 | 165 | NZ_AAMS01000002.1/215687-215778 | 92 |
| NZ_AAYC01000008.1/71109-70945 | 165 | NZ_AAPJ01000005.1/332720-332629 | 92 |
| NC_008209.1/1187650-1187815 | 166 | NC_007493.1/3168895-3168987 | 93 |
| NZ_AALY01000001.1/1161801-1161636 | 166 | NC_009428.1/2843889-2843981 | 93 |
| NZ_ABCL01000007.1/12923-13088 | 166 | NZ_AALV01000001.1/1015631-1015539 | 93 |
| NC_006677.1/1200182-1200348 | 167 | NZ_AALZ01000002.1/205814-205722 | 93 |
| NC_008380.1/2705470-2705642 | 173 | NZ_AATQ01000015.1/40504-40412 | 93 |
| NC_003062.1/1462264-1462091 | 174 | NZ_AATR01000002.1/404671-404579 | 93 |
| NC_007761.1/2340809-2340982 | 174 | NZ_ABCL01000007.1/211198-211106 | 93 |
| NZ_AAMT01000001.1/55593-55420 | 174 | NZ_AALV01000003.1/413092-412999 | 94 |
| NC_008686.1/167453-167279 | 175 | NZ_AALZ01000004.1/372786-372693 | 94 |
| NZ_AAMS01000001.1/332724-332549 | 176 | NZ_AAMO01000018.1/23903-23996 | 94 |
| NZ_AAYB01000001.1/868869-868694 | 176 | NZ_AAMU01000001.1/1377389-1377482 | 94 |
| NC_007778.1/1294499-1294677 | 179 | NZ_AAMV01000001.1/793967-794060 | 94 |
| NC_008686.1/2346604-2346426 | 179 | NZ_ABCR01000002.1/348322-348415 | 94 |
| NZ_AAOT01000056.1/1799-1977 | 179 | NC_008209.1/540401-540495 | 95 |
| NC_002679.1/179373-179552 | 180 | NZ_AAMV01000009.1/144958-144864 | 95 |
| NC_005296.1/4348220-4348399 | 180 | NZ_AAYB01000001.1/1153071-1152977 | 95 |
| NC_007958.1/1946621-1946441 | 181 | NC_002978.6/463803-463708 | 96 |
| NC_009668.1/1274930-1274750 | 181 | NC_003063.1/1761990-1761895 | 96 |
| NC_007802.1/3691970-3692151 | 182 | NC_003078.1/1192148-1192053 | 96 |
| NC_008044.1/2517447-2517265 | 183 | NC_008254.1/3522172-3522267 | 96 |
| NC_008435.1/1788927-1788745 | 183 | NC_009620.1/1477043-1476948 | 96 |
| NC_007925.1/1704052-1703869 | 184 | NZ_AAQP01000239.1/712-807 | 96 |
| NC_004463.1/6318642-6318826 | 185 | NZ_ABCL01000005.1/83069-82974 | 96 |
| NC_006933.1/503740-503925 | 186 | NC_008043.1/704802-704898 | 97 |
| NC_008358.1/2881958-2882144 | 187 | NC_008209.1/720040-720136 | 97 |
| NC_008254.1/1118967-1118778 | 190 | NC_008686.1/34903-34807 | 97 |
| NC_002678.2/707689-707879 | 191 | NZ_AAOT01000001.1/82035-82131 | 97 |
| NC_005956.1/1435974-1435783 | 192 | NZ_AAVE01000002.1/124359-124263 | 97 |
| NZ_AATH01000005.1/125179-125370 | 192 | NC_003911.11/45973-46070 | 98 |
| NC_007964.1/1820941-1820749 | 193 | NC_008044.1/2982731-2982634 | 98 |
| NZ_AANB01000010.1/45219-45025 | 195 | NC_008347.1/660403-660500 | 98 |
| NC_002696.2/3682615-3682420 | 196 | NC_009484.1/1068918-1068821 | 98 |
| NC_007406.1/1420036-1419839 | 198 | NZ_AAAP01000319.1/234-137 | 98 |
| NC_007778.1/4276466-4276663 | 198 | NZ_AAMQ01000003.1/244157-244060 | 98 |
| NC_007722.1/2045701-2045503 | 199 | NZ_AAXZ01000002.1/73598-73501 | 98 |
| NC_008242.1/148582-148780 | 199 | NZ_ABEC01000003.1/313037-312940 | 98 |
| NZ_AAMY01000007.1/127236-127434 | 199 | NZ_ABEX01000001.1/467206-467109 | 98 |
| NZ_ABAY01000238.1/6403-6204 | 200 | NC_002696.2/2234291-2234193 | 99 |
| NZ_ABCG01000003.1/324705-324904 | 200 | NC_009511.1/3517936-3518034 | 99 |
| NZ_ABEC01000010.1/152438-152639 | 202 | NZ_AAOT01000007.1/78614-78516 | 99 |
| NZ_AAPJ01000007.1/84897-85099 | 203 | NC_003317.1/1774842-1774941 | 100 |
| NC_008343.1/2635664-2635867 | 204 | NC_006932.1/229609-229510 | 100 |
| NZ_AATH01000001.1/1914714-1914510 | 205 | NC_007794.1/1926155-1926254 | 100 |
| NZ_AATP01000001.1/743446-743650 | 205 | NZ_AATH01000001.1/1480912-1480813 | 100 |
| NC_009445.1/5178587-5178792 | 206 | NZ_AAUW01000002.1/180081-179982 | 100 |
| NC_009484.1/49216-49421 | 206 | NZ_AAAP01001729.1/337-237 | 101 |
| NC_009485.1/5669949-5670154 | 206 | NZ_ABAY01000007.1/48650-48750 | 101 |
| NC_009511.1/2979106-2979311 | 206 | NC_007722.1/1989274-1989173 | 102 |
| NC_002696.2/3603520-3603314 | 207 | NZ_AAMT01000023.1/22963-23064 | 102 |
| NC_008048.1/1968004-1967796 | 209 | NZ_AATP01000003.1/96900-96799 | 102 |
| NZ_AAMW01000002.1/63372-63161 | 212 | NZ_ABCG01000003.1/174756-174857 | 102 |
| NZ_AAQG01000010.1/114332-114121 | 212 | NC_006526.1/155239-155341 | 103 |
| NZ_AAMQ01000006.1/12559-12771 | 213 | NC_003062.1/2700139-2700036 | 104 |
| NZ_AAMU01000001.1/1311427-1311639 | 213 | NC_009485.1/6469821-6469924 | 104 |
| NZ_AAUW01000010.1/155024-155236 | 213 | NZ_AAQG01000016.1/58799-58696 | 104 |
| NC_007626.1/822411-822628 | 218 | NZ_AAUW01000003.1/247167-247064 | 104 |
| NC_008347.1/2414251-2414034 | 218 | NZ_AAYA01000001.1/176626-176729 | 104 |
| NC_009719.1/749158-749375 | 218 | NC_003062.1/2541734-2541630 | 105 |
| NZ_AAAP01003811.1/3058-2840 | 219 | NC_003078.1/1633734-1633838 | 105 |
| NC_009720.1/2461338-2461117 | 222 | NC_008048.1/2492584-2492688 | 105 |
| NC_007794.1/1949623-1949846 | 224 | NC_009620.1/649479-649375 | 105 |
| NC_007643.1/3515512-3515283 | 230 | NZ_AAMT01000008.1/131819-131715 | 105 |
| **Mean glycine riboswitch length (nt)** | **187.3** | NC_007626.1/254095-253990 | 106 |
| **Standard deviation** | **20.0** | NC_008381.1/146980-146875 | 106 |
|  |  | NC_009719.1/2328672-2328777 | 106 |
|  |  | NZ_AAAP01003877.1/60656-60551 | 106 |
| **SRP RNA** |  | NZ_AAMW01000002.1/15994-16099 | 106 |
| **RefSeqAccession** | **Length** | NZ_AATP01000001.1/52615-52720 | 106 |
| NC_007798.1/47944-48025 | 82 | NZ_AATR01000053.1/3103-3208 | 106 |
| NZ_ABCG01000009.1/1329-1414 | 86 | NZ_AAYB01000002.1/825436-825541 | 106 |
| NZ_AAFF01000001.1/1138225-1138316 | 92 | NC_002678.2/1469030-1468924 | 107 |
| NC_002696.2/281948-282040 | 93 | NC_003047.1/3532865-3532971 | 107 |
| NC_006831.1/1485490-1485583 | 94 | NC_007761.1/4094591-4094697 | 107 |
| NZ_AAIT01000003.1/182719-182812 | 94 | NC_008380.1/4678113-4678219 | 107 |
| NC_007797.1/1413055-1412962 | 94 | NC_002678.2/3104122-3104015 | 108 |
| NC_007618.1/1473116-1473023 | 94 | NC_009636.1/3252666-3252773 | 108 |
| NC_007794.1/1403600-1403695 | 96 | NC_009667.1/1203765-1203658 | 108 |
| NZ_AAYA01000009.1/121008-120912 | 97 | NZ_AALY01000003.1/280470-280577 | 108 |
| NZ_AAQG01000012.1/70329-70425 | 97 | NC_007802.1/336841-336733 | 109 |
| NC_007799.1/11848-11945 | 98 | NC_002678.2/4674022-4673913 | 110 |
| NC_004842.2/1132364-1132267 | 98 | NZ_AAMO01000007.1/212652-212543 | 110 |
| NC_009445.1/808247-808344 | 98 | NC_008254.1/2750090-2750200 | 111 |
| NC_007964.1/511489-511586 | 98 | NZ_AAMO01000007.1/207774-207885 | 112 |
| NZ_AAFE01000001.1/1203944-1204041 | 98 | NZ_AALY01000003.1/286702-286815 | 114 |
| NC_009485.1/7712163-7712066 | 98 | NZ_AAUW01000008.1/143415-143528 | 114 |
| NC_005296.1/682367-682464 | 98 | NZ_AAMV01000007.1/209951-209837 | 115 |
| NC_003062.1/101343-101246 | 98 | NC_008044.1/2156715-2156830 | 116 |
| NC_006677.1/645681-645584 | 98 | NZ_AAAP01002426.1/1018-1133 | 116 |
| NC_007354.1/1309520-1309617 | 98 | NZ_AAVE01000007.1/53853-53738 | 116 |
| NC_008048.1/801533-801631 | 99 | NZ_ABAY01000257.1/152-267 | 116 |
| NZ_AAXZ01000002.1/600191-600289 | 99 | NZ_ABEC01000011.1/105364-105479 | 116 |
| NZ_AAYB01000001.1/327962-328060 | 99 | NZ_ABEX01000017.1/145204-145319 | 116 |
| NC_009511.1/53278-53376 | 99 | NC_008343.1/114911-115028 | 118 |
| NZ_AALZ01000001.1/399587-399685 | 99 | NZ_AAPJ01000006.1/130400-130282 | 119 |
| NZ_AAMU01000001.1/712702-712604 | 99 | NZ_AANB01000004.1/39728-39847 | 120 |
| **NC_007205.1/493567-493665** | **99** | NZ_AAYC01000010.1/114871-114752 | 120 |
| NZ_AAPV01000001.1/397870-397772 | 99 | NZ_ABAY01000501.1/1991-2110 | 120 |
| NC_005955.1/281771-281673 | 99 | NZ_AAAP01002670.1/1241-1361 | 121 |
| NC_005956.1/309929-309831 | 99 | NZ_AAPJ01000011.1/75144-75264 | 121 |
| NC_006932.1/40620-40522 | 99 | NZ_AATQ01000077.1/11950-11830 | 121 |
| NC_009667.1/21485-21583 | 99 | NC_004463.1/7330682-7330803 | 122 |
| NC_003047.1/259929-260027 | 99 | NC_006677.1/1475626-1475504 | 123 |
| NC_003103.1/1239527-1239625 | 99 | NC_008687.1/111129-111252 | 124 |
| NC_007109.1/1454207-1454305 | 99 | NC_007763.1/93916-93792 | 125 |
| NC_006526.1/868953-869051 | 99 | NC_008687.1/822667-822791 | 125 |
| NZ_ABCR01000016.1/27807-27708 | 100 | NZ_ABCL01000002.1/442429-442305 | 125 |
| NZ_AAMS01000006.1/209424-209523 | 100 | NC_007643.1/2319857-2319982 | 126 |
| NZ_ABEX01000001.1/121672-121771 | 100 | NC_009720.1/3914689-3914815 | 127 |
| NZ_AATQ01000010.1/20630-20531 | 100 | NZ_ABCR01000001.1/235709-235583 | 127 |
| NZ_ABAY01000001.1/47727-47826 | 100 | NC_006677.1/2443327-2443454 | 128 |
| NC_009049.1/2502810-2502909 | 100 | NC_007406.1/2687699-2687826 | 128 |
| NZ_AAOT01000027.1/51084-51183 | 100 | NC_007964.1/3185661-3185789 | 129 |
| NZ_AATR01000003.1/10486-10585 | 100 | NZ_AAMY01000014.1/40007-40135 | 129 |
| NZ_AAAP01003231.1/1453-1552 | 100 | NC_007925.1/2359237-2359108 | 130 |
| NC_008380.1/166490-166391 | 100 | NC_005296.1/4039380-4039511 | 132 |
| NZ_AANB01000018.1/31767-31668 | 100 | NC_007958.1/3812110-3812244 | 135 |
| NZ_AAVE01000004.1/254857-254758 | 100 | NC_008435.1/2282986-2282852 | 135 |
| NC_007626.1/4656380-4656479 | 100 | NC_007778.1/2225706-2225564 | 143 |
| NC_008358.1/888166-888265 | 100 | NC_009667.1/296481-296332 | 150 |
| NZ_AATH01000010.1/64487-64388 | 100 | NC_006932.1/1705893-1706056 | 164 |
| NZ_AAGB01000288.1/1257-1158 | 100 | **Mean TPP riboswitch length (nt)** | **112.9** |
| NZ_AATR01000042.1/33-132 | 100 | **Stdev** | **12.7** |
| NC_003911.11/3754764-3754863 | 100 |  |  |
| NC_002978.6/1020406-1020505 | 100 |  |  |
| NC_008347.1/512363-512463 | 101 | **RNaseP** |  |
| NC_007643.1/3998696-3998596 | 101 | **RefSeqAccession** | **Length** |
| NC_009719.1/276641-276741 | 101 | NC_004842.2/887251-887582 | 332 |
| NC_009484.1/2193808-2193708 | 101 | NC_007354.1/922327-922662 | 336 |
| NC_008254.1/4368685-4368585 | 101 | NC_006832.1/1072905-1073242 | 338 |
| NC_009720.1/2430563-2430463 | 101 | NC_007797.1/215238-214901 | 338 |
| NC_009636.1/3636444-3636544 | 101 | NZ_AAIF01000004.1/23278-22941 | 338 |
| NZ_AATP01000001.1/106899-106999 | 101 | NC_006833.1/780822-781168 | 347 |
| NZ_AAUW01000009.1/200222-200322 | 101 | NZ_AATR01000004.1/214622-214276 | 347 |
| NZ_AAPJ01000004.1/104994-105094 | 101 | NC_002978.6/707034-706687 | 348 |
| NZ_AABW01000001.1/696905-696805 | 101 | NZ_AAGC01000004.1/5657-5310 | 348 |
| NZ_AAVR01000001.1/1338873-1338773 | 101 | NZ_AAQP01000136.1/1304-1651 | 348 |
| NZ_AAGC01000158.1/803-903 | 101 | **NZ_AAPV01000001.1/853324-853678** | **355** |
| NC_006833.1/565558-565458 | 101 | NZ_ABAY01000330.1/4837-4470 | 368 |
| NZ_AAMT01000005.1/227966-227865 | 102 | NC_006142.1/609189-608806 | 384 |
| NZ_ABCL01000006.1/51250-51149 | 102 | NC_009488.1/1549288-1548904 | 385 |
| NZ_AALY01000003.1/35449-35348 | 102 | NC_005956.1/1251235-1250846 | 390 |
| NZ_AAMO01000003.1/50121-50020 | 102 | NC_005955.1/1054739-1054348 | 392 |
| NC_008209.1/1218334-1218233 | 102 | NC_007940.1/1294492-1294098 | 395 |
| NC_008044.1/185752-185651 | 102 | NC_009428.1/2259552-2259157 | 396 |
| NZ_AAYC01000008.1/27834-27733 | 102 | NC_003047.1/2357178-2356781 | 398 |
| NC_007802.1/3970797-3970898 | 102 | NC_008380.1/3479602-3479205 | 398 |
| NZ_AAMQ01000002.1/459408-459307 | 102 | NC_009049.1/974369-974766 | 398 |
| NC_007940.1/49453-49554 | 102 | NC_003062.1/2073734-2073333 | 402 |
| NC_009488.1/1840698-1840597 | 102 | NZ_AAAP01003400.1/1456-1054 | 403 |
| NC_002678.2/4414153-4414254 | 102 | NZ_ABEX01000012.1/38232-38635 | 404 |
| NC_008783.1/1281191-1281293 | 103 | NZ_AAAP01003643.1/1326-1732 | 407 |
| NZ_AALV01000001.1/456292-456394 | 103 | NZ_AALV01000011.1/14272-14683 | 412 |
| NC_008343.1/307224-307122 | 103 | NC_009445.1/5866896-5867321 | 426 |
| NZ_AAMW01000001.1/567228-567330 | 103 | NC_009505.1/1409249-1408824 | 426 |
| NC_000963.1/1090690-1090792 | 103 | NC_008435.1/2310384-2309954 | 431 |
| NC_006142.1/1090175-1090277 | 103 | NC_007778.1/2253459-2253028 | 432 |
| **Mean SRP (4.5S) RNA length (nt)** | **99.3** | NZ_AAMY01000014.1/32739-33188 | 450 |
| **Stdev** | **3.3** | NC_005296.1/4004668-4005149 | 482 |
|  |  | NC_007109.1/799267-799765 | 499 |
|  |  | NC_003103.1/739871-739357 | 515 |
|  |  | NZ_AADJ01000001.1/666733-667249 | 517 |
|  |  | NZ_AAUY01000001.1/662029-662545 | 517 |
|  |  | NZ_AABW01000001.1/12286-11769 | 518 |
|  |  | NZ_AAVR01000001.1/680077-680596 | 520 |
|  |  | **Mean RNaseP RNA length (nt)** | **406.3** |
|  |  | **Stdev** | **58.8** |
